# Adult Langerhans cell histiocytosis presenting with multisystem involvement and sarcomatoid features: a case report

**DOI:** 10.1186/s13256-020-02460-3

**Published:** 2020-09-27

**Authors:** Luis E. Aguirre, Ingrid Schwartz, Jennifer Chapman, Marcelo F. Larsen, Alvaro Alencar

**Affiliations:** 1 Department of Hematology and Medical Oncology, Moffitt Cancer Center and Research Institute, Tampa, FL USA; 2 Department of Gastroenterology, Loyola University Medical Center, Maywood, IL USA; 3 Department of Pathology, University of Miami Miller School of Medicine, Miami, FL USA; 4 Department of Gastroenterology, University of Miami Miller School of Medicine, Miami, FL USA; 5 Department of Hematology/Oncology, Sylvester Comprehensive Cancer Center, University of Miami Miller School of Medicine, Clinical Research Building, 1120 NW 14th Street, Suite 650B, Miami, FL 33136 USA

**Keywords:** Langerhans cell histiocytosis, Langerhans cell sarcoma, Cytogenetics, Acute myeloid leukemia, Colony-stimulating factor, Leukemic transformation, Chemotherapy, Central nervous system

## Abstract

**Background:**

Langerhans cell tumors are rare clonal disorders characterized by neoplastic proliferation of dendritic cells that can be further classified into the subtypes Langerhans cell histiocytosis and Langerhans cell sarcoma, which are rare neoplasms exhibiting aggressive features and a poor prognosis. In addition to illustrating the refractoriness and poor outcomes of multisystem Langerhans cell histiocytosis in adults, specific events in this case highlight important characteristics of disease biology that warrant detailed discussion and exposition to a wider audience.

**Case presentation:**

We describe the case of a 42-year-old Caucasian man with Langerhans cell histiocytosis diagnosed from a lesion on the left arm that presented with constitutional symptoms, early satiety, and weight loss. Esophagogastroduodenoscopy showed extensive esophageal and duodenal involvement by Langerhans cell histiocytosis with features of Langerhans cell sarcoma. He was initially treated for Langerhans cell histiocytosis with low doses of cytarabine until he eventually presented clear transformation to acute monoblastic leukemia with complex karyotype that could not be properly controlled, leading eventually to death.

**Conclusions:**

Langerhans cell histiocytosis remains an exceedingly rare entity in adults, frequently presenting as multisystem disease with risk organ involvement. Langerhans cell sarcoma represents an aggressive subtype with extremely poor prognosis for which intensive acute myeloid leukemia induction should be strongly considered.

## Background

Langerhans cell tumors are rare clonal disorders characterized by neoplastic proliferation of dendritic cells with distinctive protein expression (CD1a/langerin/S100) and ultrastructural features (Birbeck granules) with Langerhans cell histiocytosis (LCH) and Langerhans cell sarcoma (LCS) subtypes [1–3]. Historically, LCH has been classified as a unifocal disease, multifocal unisystem disease, or multifocal multisystem disease [2]. LCS is a rare neoplasm exhibiting aggressive features that carries a poor prognosis. We report a case of a patient with a presentation with symptomatic multisystem multifocal LCH disease with early suggestion of leukemic transformation with particular sensitivity to growth factors. In addition to illustrating the refractoriness and poor outcomes of multisystem LCH in adults, specific events in this case highlight important characteristics of disease biology that warrant detailed discussion and exposition to a wider audience to raise awareness of this unusual entity and aid in the earlier identification of its potential complications.

## Case presentation

**Fig. 1:**
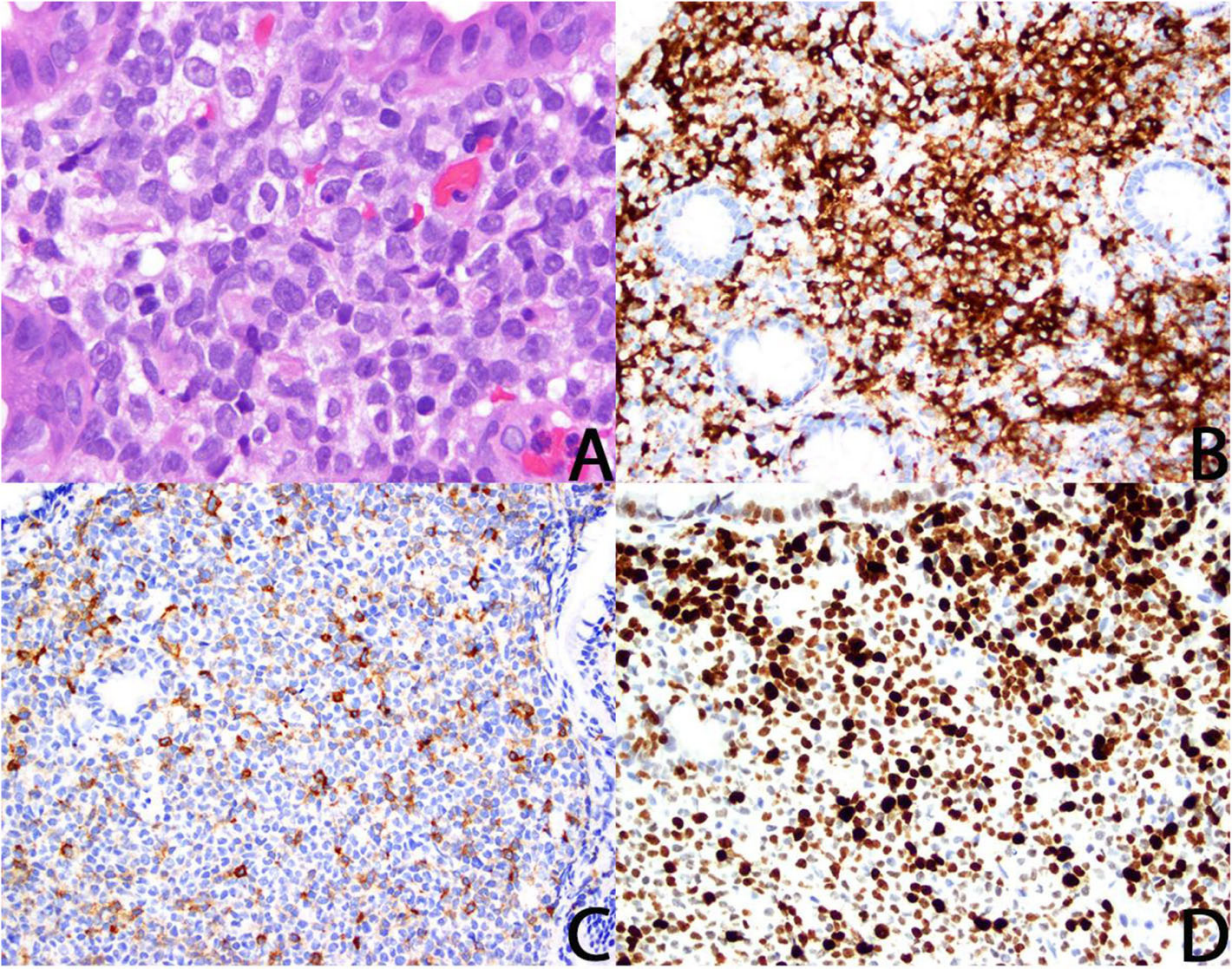
Langerhans cell histiocytosis with progression to Langerhans cell sarcoma involving gastric and small intestine mucosa. Endoscopically derived biopsies of gastric and small intestine mucosa showed involvement by Langerhans cell histiocytosis. Pronounced pleomorphism, apoptosis, and mitotic figures were frequent, consistent with progression to Langerhans cell sarcoma (**a**; H&E stain, 1000× magnification). Neoplastic cells expressed langerin protein (**b**; 400× magnification), and a subset showed preserved expression of CD1a (**c**; 400× magnification). Langerhans cells were negative for S100. Ki67-based proliferative rate was estimated at 85% (**d**; 400× magnification). Although objective distinction between involvement by systemic Langerhans cell histiocytosis and Langerhans cell sarcoma is difficult, the pleomorphic features of the neoplastic cells, aberrant diminished expression of CD1a and S100, and high proliferative rate support designation as Langerhans cell sarcoma. The result of B-RAF testing was negative

A 42-year-old Caucasian man presented to our hospital with acute onset of fatigue, nausea, vomiting, early satiety, diarrhea, and weight loss 3 months after LCH had been diagnosed on the basis of biopsy of an asymptomatic lesion on the left arm. His physical examination was pertinent for tender hepatosplenomegaly and multiple eczematous papular lesions on the trunk and extremities. His complete blood count (CBC) results and lactate dehydrogenase (LDH) level were normal. Esophagogastroduodenoscopy (EGD) demonstrated herpes esophagitis and *Helicobacter pylori* gastritis. Biopsies of the duodenum and esophagus showed involvement by LCH with features suggestive of LCS (Fig. 1). The *BRAF V600E* mutation was not detected. Imaging did not demonstrate any suspicious bone or central nervous system (CNS) involvement. His bone marrow biopsy (BMBx) showed no LCH or acute myeloid leukemia (AML). He was started on intravenous cytarabine 100 mg/m^2^ daily for 5 days every 4 weeks, which led to improvement but no resolution of his skin lesions or gastrointestinal (GI) symptoms. After four cycles, his therapy was adjusted to every 2 weeks in view of kinetic failure. This adjustment led to clinical improvement, but his LDH level continued to rise, leading to repeat BMBx performed after cycle 8, which showed no evidence of AML.

**Fig. 2:**
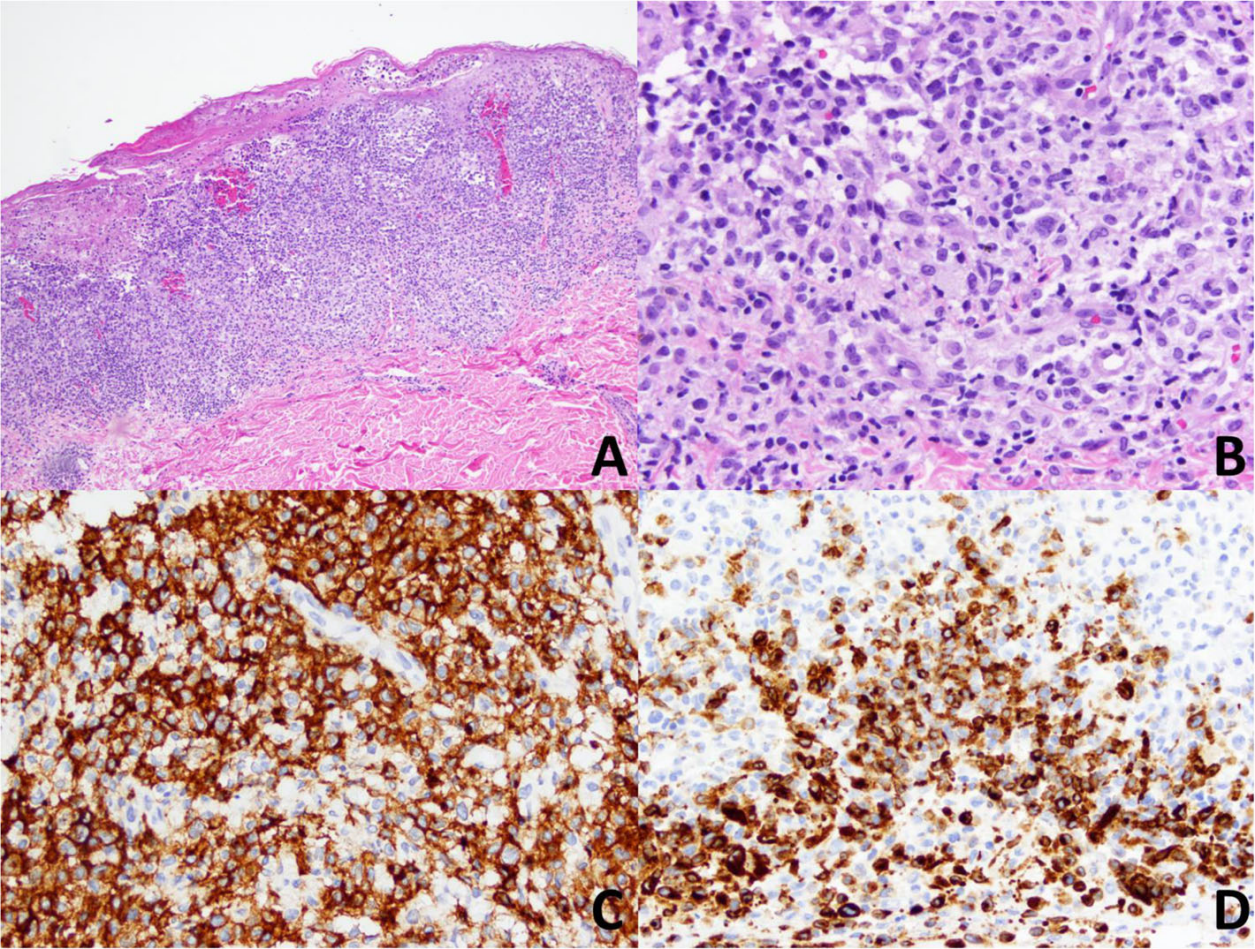
Punch biopsy of the skin showing superficial epidermal involvement by a hematopoietic neoplasm. Epidermal erosion and ulceration with underlying hematopoietic infiltrate involving epidermis and upper dermis (**a**; H&E stain, 100× magnification). The hematopoietic neoplasm is poorly differentiated with blastic nuclear features and round to reniform nuclei. Cellular apoptosis is increased. Numerous small reactive lymphoid cells are also present (**b**; 500× magnification). The majority of the blastic cells express CD1a by immunohistochemistry (**c**; 500× magnification) and coexpress langerin (**d**; 500× magnification). The findings are those of acute monoblastic leukemia with Langerhans cell differentiation involving skin

Three weeks later, he presented with acute left hemianopia resulting from an ischemic cerebrovascular accident. Workup demonstrated circulating blasts, spontaneous tumor lysis syndrome, and disseminated intravascular coagulation (DIC). BMBx confirmed acute monoblastic leukemia with a complex karyotype. Next-generation sequencing (NGS) showed no additional mutations. The patient was started on induction 7 + 3 (daunorubicin/cytarabine), which led to transient resolution of skin lesions that quickly worsened by day 15. The lesions appeared as multiple erythematous papules and nodules throughout the back. Histological sections showed heavily epidermotropic and bandlike dermal infiltrates of leukemic cells in the dermis with pseudo-blisters formed by tumor necrosis (Fig. 2a). A subset of tumor cells showed features of Langerhans cells, including reniform nuclei and atypical large and hyperchromic nuclei (Fig. 2b). Immunophenotyping revealed that the tumor cells were positive for langerin, S100, and CD1a with intratumoral heterogeneity and a Ki67 showing a nearly 80% cell proliferation rate (Fig. 2c, d). The tumor cells were also positive for CD56, CD117, and CD123 in a subset of cells. The immunophenotype supported both myelomonoblastic and Langerhans cell differentiation. Marrow cytogenetics detected t(13:14) in all tumor cells and additional structural abnormalities involving chromosomes 1, 8, 9, 10, and Y. In this particular context, the cutaneous lesions were best classified as cutaneous involvement of leukemia. Cytogenetic profiling with NGS showed nonsynonymous mutation p.E69K affecting the *PTPN11* gene (SHP2), homozygous loss of *CDKN2A* at 9p21, and a tumor mutational burden of 26%.

**Fig. 3:**
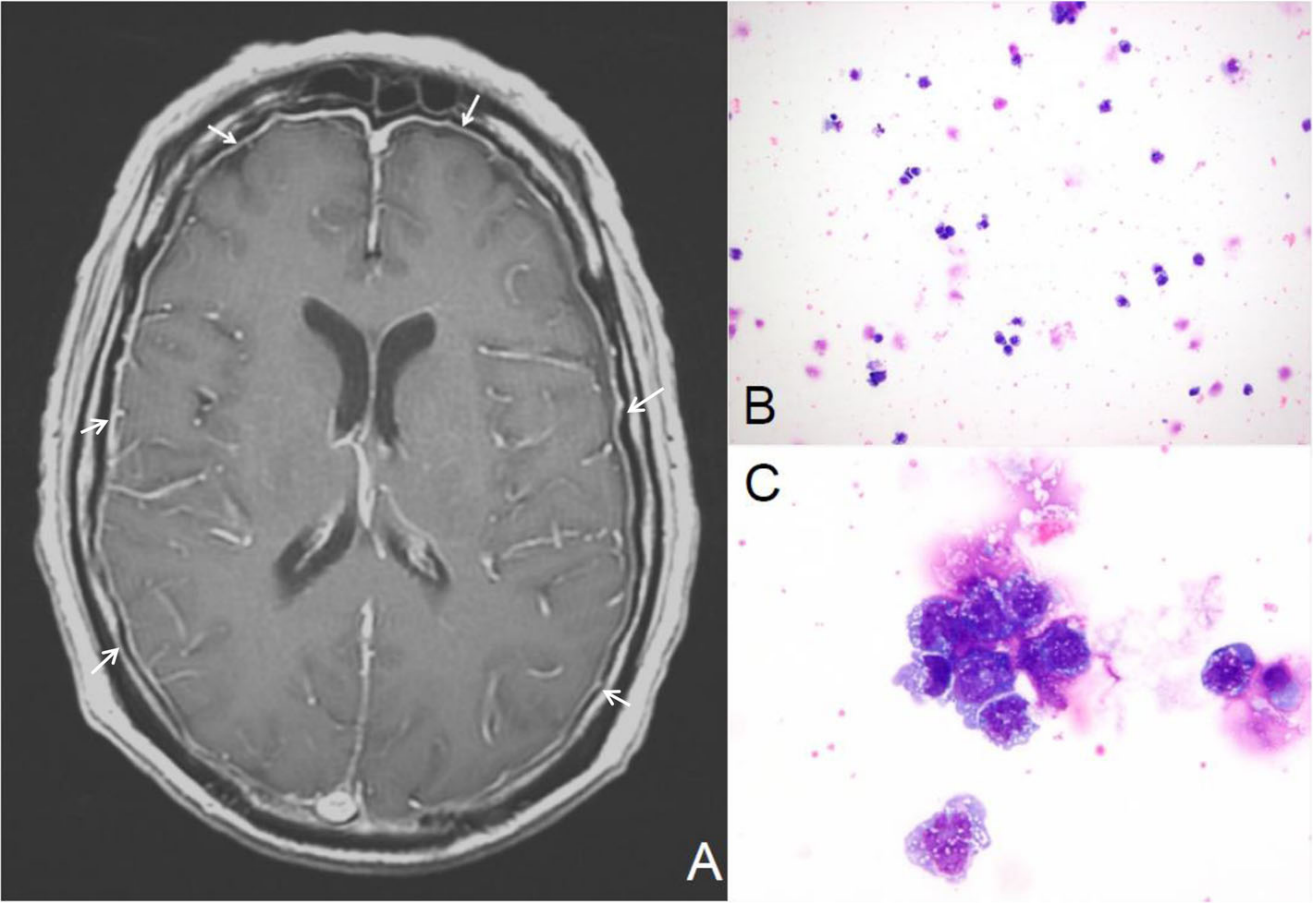
T1-weighted magnetic resonance imaging (MRI) of the brain with contrast enhancement (sagittal view) and cerebrospinal fluid analysis. Leptomeningeal involvement by acute myeloid leukemia demonstrated by brain MRI with contrast (**a**) with diffuse pachymeningeal thickening and enhancement overlying the bilateral cerebral hemispheres (*white arrows*) and cerebrospinal fluid showing abnormal hypercellularity due to the presence of monoblasts (**b** and **c**; Wright Giemsa stain at 200× and 1000× magnification, respectively)

He was started on salvage therapy with CLAG-M (cladribine 5 g/m^2^, cytarabine 2 g/m^2^, granulocyte colony-stimulating factor (G-CSF), mitoxantrone 10 g/m^2^), and after the first dose of filgrastim (G-CSF), he developed acute diplopia with leptomeningeal involvement by AML as identified by brain magnetic resonance imaging and lumbar puncture (Fig. 3). Intensive intrathecal therapy with methotrexate (MTX) was initiated twice per week for six cycles. The patient’s skin lesions and neurologic symptoms resolved with restaging consistent with complete remission. However, 1 week later, his skin lesions recurred, followed by recurrence of neurologic deficits that responded poorly to salvage therapy, including high-dose cytarabine, further intrathecal therapy, and CNS radiation. The patient died 1 year after diagnosis of LCH while undergoing treatment with MTX and cytarabine as a bridge to marrow transplant. Table 1 summarizes the cardinal features associated with this case.

**Table 1:** Oncologic history/summary

January 2017: Skin biopsy showed LCH	
February 2017: Progressive nausea/diarrhea, weight loss	
March 2017: EGD - herpes esophagitis, Barrett’s esophagus, *Helicobacter pylori* gastritis, Langerhans cell sarcoma in stomach/duodenum Acyclovir full dose, triple therapy (could not tolerate)	
April 2017: Persistent nausea/emesis, weight loss EGD: Resolved herpes esophagitis, Barrett’s esophagus, persistent gastritis, Langerhans cell sarcoma in stomach/duodenum PET/CT: Diffuse uptake in bone marrow, and GI tract Brain MRI (−) Bone MRI: Diffuse marrow changes, no bone lesions Normal CBC, LDH BMBx (−)	
May 2017: Cytarabine 100 mg/m2/d × 5 d	
July 2017: EGD/colonoscopy post C3 - still significant erythema, biopsy (+)	
August 2017: Improved but persistent symptoms Skin biopsy - LCH	
September 2017: C6 - switch to every 2 weeks	
October 2017: BMBx post C7 – No evidence of disease, dyspoiesis likely from chemo, (−) AML FISH and myeloid NGS	
November 2017: Right posterior cerebral artery [PCA] thrombus s/p TPA Circulating blasts: BMBx - acute monoblastic leukemia Karyotype: 45,XY,der(13;14)(q10;q10) 45,X,der(Y)t(Y;?)(q11.2;?), sl, der(1)t(1;9)(p10;q10), der(8)t(1;8)(q10;p10), del(10)(p11.2) AML NGS (−), FISH (−)	
November 2017: 7 + 3 - D15: Recurrent skin lesions BMBx - persistent AML Karyotype: 45,XY,der(13;14)(q10;q10) Skin lesion: Leukemia cutis with LCH (+) AML NGS PTPN11 mutation FISH (+) homozygous del 9p21	
December 2017: CLAG-M - diplopia on D1 (+) MRI and LP for CSF involvement LP with IT chemo 2x/week × 6 - (−) first LP Recovery marrow - CR	
January 2018: HiDAC 3 g/m^2^ x C1 + IT	
February 2018: Cytarabine 2 g/m^2^ + cladribine 5 mg/m^2^ + mitoxantrone 10 mg/m^2^ + IT	
March 2018: MTX 5 g/m^2^ + cytarabine 3 g/m^2^ as a bridge to transplant due to new skin lesions + IT	

*AML* acute myeloid leukemia, *BMBx* bone marrow biopsy, *CBC* complete blood count, *C#* cycle number, *CLAG-M* cladribine, cytarabine, granulocyte colony stimulating factor, mitoxantrone, *CR* complete response, *D#* day number, *EGD* esophagogastroduodenoscopy, *FISH* fluorescent in situ hybridization, *GI* gastrointestinal, *HiDAC* high-dose cytarabine, *IT* intrathecal, *LCH* Langerhans cell histiocytosis, *LDH* lactate dehydrogenase, *LP* lumbar puncture, *MRI* magnetic resonance imaging, *MTX* methotrexate, *NGS* next generation sequencing, *PTPN11* Protein tyrosine phosphatase, non-receptor type 11, *TPA* tissue plasminogen activator

## Discussion and conclusions

LCH is a rare entity that results from atypical clonal proliferation of a subset of mononuclear dendritic cells closely resembling Langerhans cells that derive from myeloid progenitor cells. Somatic mutations activating the MAPK (mitogen-activated protein kinase) signaling pathway are commonly observed in LCH, with *BRAF V600E* mutation identified in over half cases [6, 7]. LCH is mostly described and studied in children [1]. Median age at the time of diagnosis is 1–3 years with an annual incidence of 3-5 cases per million persons per year in children and 1-2 cases per million persons per year in adults [2, 3] LCH is further stratified into single-system disease with unifocal or multifocal involvement or multifocal multisystem disease with or without risk organ (marrow, liver, or spleen) involvement [1–3]. The majority of adults present with multisystem involvement mostly characterized by osteolytic lesions, skin or mucocutaneous involvement, diabetes insipidus, and hepatosplenomegaly. CNS or GI compromise is uncommon [8–10]. Prognosis correlates with extent of disease and degree of organ dysfunction, with more than half presentations with multisystem disease dying of LCH [4, 11]. LCS, reported mainly in adults, is extremely rare. Differentiation from LCH is challenging because immunophenotype is identical and diagnosis is based on overtly malignant pleomorphic appearance [11–15].

Therapy is extrapolated from pediatric studies and single-institution experiences because clinical trials in adults are lacking [16]. Although the commonly used pediatric approach with vinblastine and prednisone is acceptable, single-agent cytarabine or cladribine has been the preferred first-line option, mostly to minimize steroid-induced toxicity and neuropathy [17]. A review of the management of 58 adults with LCH demonstrated significantly higher response with cytarabine than with vinblastine/prednisone, which also presented worse toxicity with 75% grade 3–4 neuropathy [18]. Recurrences in adults are common. Besides the use of other cytotoxic agents such as clofarabine, the BRAF inhibitor vemurafenib has demonstrated excellent, albeit transient, responses in patients with *BRAF V600E mutation* [19]. The role of consolidation with allogeneic stem cell transplant is unclear. A review of 87 patients with high-risk LCH who underwent allogeneic transplant demonstrated greater than 70% long-term survival with higher relapse rates with reduced-intensity conditioning [12, 20]. A literature review of LCS described dramatic variation in management from chemotherapy to surgery, radiation, or a combination of them. Chemotherapy regimens were CHOP (cyclophosphamide, doxorubicin, vincristine, and prednisone)-based with mixed results. Survival was overall poor, 58% at 1 year, and outcomes were poorer in disseminated presentations [12].

Our case highlights the refractoriness of LCH in adults. Agents known to have activity in LCH demonstrated suboptimal responses. Attention to specific events during our patient’s presentation highlight important biologic characteristics of LCH. Molecular therapy was not possible, because no targetable mutation was identified. One central finding was the detection of LCS by EGD biopsy. Although our patient presented with symptomatic multisystem disease that required systemic therapy, the LCS component was minute. A more intensive first-line therapy was contemplated but was believed not to be indicated, considering that the presentation was mostly of LCH with a negligible LCS component with normal CBC, LDH, and BMBx. The best response was achieved after an AML induction/salvage regimen (cladribine, cytarabine, and filgrastim with idarubicin [CLAG-Ida]) that was intentionally chosen in view of the described activity of cladribine in LCH [5, 21]. One is left to wonder if our patient’s outcome would have been different had he received intensive induction chemotherapy for what was ultimately a presentation of myeloid sarcoma.

CNS involvement was another critical event. Although our patient had no suggestion of CNS involvement at initial staging, an ischemic cerebrovascular accident believed to be closely related to the DIC was the hallmark of presentation at disease progression/transformation to AML. Nonetheless, the most intriguing aspect of CNS involvement was the acute development of diplopia after the first dose of G-CSF administered as part of CLAG-Ida. The potential relationship between CNS involvement and G-CSF was highlighted during salvage, when the resolved diplopia recurred after a dose of pegfilgrastim. Numerous serum cytokines, including G-CSF, have been associated with the pathogenesis of LCH [22, 23]. The timing between growth factor injection and development of neurologic deficits implies a potential correlation.

The homozygous loss of *CDKN2A* at 9p21 was also an interesting finding when analyzing the cytogenetic profile of our patient’s tumor. In contrast to its counterpart of LCH, a recent study demonstrated frequent homozygous loss of *CDKN2A/B* locus (9p21) and both *MAP2K1* and *NRAS* genes mutations in LCS, which may provide a future basis for potential targeted therapy [24].

In summary, LCH remains an exceedingly rare entity in adults, frequently presenting as multisystem disease with risk organ involvement. Although therapeutic targets such as *BRAF V600E* mutations have been identified, therapeutic options remain limited. An intensive AML induction regimen should be strongly considered for LCH presentations with suggestion of an LCS component with cautious use of growth factors and particular attention to CNS involvement. In view of the limited number of effective therapeutic interventions, patients with refractory cases or atypical presentations of LCH and LCH-related disorders should be encouraged to enroll in one of multiple active clinical trials (Table 2).

**Table 2:** Active clinical trials recruiting patients with Langerhans cell histiocytosis and related disorders

Study title (ClinicalTrials.gov identifier)	Interventions	Study design	Locations
AraC for Newly Diagnosed Adult Langerhans Cell Histiocytosis (NCT04121819)	Drug: Cytarabine	Intervention model: Single-group assignment Masking: None (open label) Primary purpose: Treatment	Peking Union Medical College Hospital, Beijing, China
Study of Clofarabine in Patients With Recurrent or Refractory Langerhans Cell Histiocytosis and LCH-related Disorders (NCT02425904)	Drug: Clofarabine	Allocation: Nonrandomized Intervention model: Single- group assignment Masking: None (open label) Primary purpose: Treatment	Phoenix Children’s Hospital, Phoenix, AZ, USA Arkansas Children’s Hospital, Little Rock, AR, USA Children’s Hospital of Los Angeles, Los Angeles, CA, USA (and 12 more)
LCH-IV, International Collaborative Treatment Protocol for Children and Adolescents With Langerhans Cell Histiocytosis (NCT02205762)	Drug: Prednisone Drug: Vinblastine Drug: Mercaptopurine (and 6 more)	Allocation: Randomized intervention model: Parallel assignment Masking: None (open label) Primary purpose: Treatment	Children’s Hospital of Alabama, Birmingham, AL, USA Phoenix Children’s Hospital, Phoenix, AZ, USA Arkansas Children’s Hospital, Little Rock, AR, USA (and 28 more)
Thalidomide, Cyclophosphamide and Dexamethasone for Recurrent/Refractory Adult Langerhans Cell Histiocytosis (NCT04120519)	Drug: Thalidomide combined with dexamethasone and cyclophosphamide	Intervention model: Single-group assignment Masking: None (open label) Primary purpose: Treatment	Peking Union Medical College Hospital, Beijing, China
Vinblastine/Prednisone Versus Single Therapy With Cytarabine for Langerhans Cell Histiocytosis (LCH) (NCT02670707)	Drug: Cytarabine Drug: Vinblastine/prednisone	Allocation: Randomized intervention model: Parallel assignment masking: None (open label) Primary purpose: Treatment	Texas Children’s Hospital, Houston, TX, USA
Denosumab for the Treatment of Adult LCH (NCT03270020)	Drug: Denosumab 70 mg/ml (XGEVA; Amgen, Charlotte, NC, USA)	Intervention model: Single-group assignment Masking: None (open label) Primary purpose: Treatment	251 Hellenic Air Force and VA Athens General Hospital, Department of Endocrinology, Attiki, Greece
Cobimetinib in Refractory Langerhans Cell Histiocytosis (LCH), and Other Histiocytic Disorders (NCT04079179)	Drug: Cobimetinib	Allocation: Nonrandomized intervention model: Parallel assignment Masking: None (open label) Primary purpose: Treatment	NACHO Consortium, Memphis, TN, USA Texas Children’s Hospital, Houston, TX, USA
A Combination of Vemurafenib, Cytarabine and 2-chlorodeoxyadenosine in Children With LCH and BRAF V600E Mutation (NCT03585686)	Drug: Vemurafenib Drug: Cytarabine Drug: 2-Chlorodeoxyadenosine	Intervention model: Single-group assignment Masking: None (open label) Primary purpose: Treatment	Dmitry Rogachev National Research Center of Pediatric Hematology, Oncology and Immunology, Moscow, Russian Federation
Inherited Genetic Susceptibility in Langerhans Cell Histiocytosis (LCH) (NCT04100408)	Other: Biospecimen collection Other: Laboratory biomarker analysis Other: Questionnaire administration	Observational model: Family-based Time perspective: Prospective	Baylor College of Medicine/Dan L. Duncan Comprehensive Cancer Center at Baylor St. Luke’s Medical Center, Houston, TX, USA
A Study of Memory, Thinking, and Brain Imaging in Adults With Histiocytosis (NCT03127709)	Behavioral: Trail Making Test parts A and B Behavioral: Brief Test of Attention Behavioral: Symbol Span (and 8 more)	Observational model: Cohort Time perspective: Prospective	Memorial Sloan Kettering Basking Ridge, Basking Ridge, NJ, USA Memorial Sloan Kettering Monmouth, Middletown, NJ, USA Memorial Sloan Kettering Cancer Center @ Suffolk, Commack, NY, USA (and 1 more)
Targeted Therapy Directed by Genetic Testing in Treating Pediatric Patients With Relapsed or Refractory Advanced Solid Tumors, Non-Hodgkin Lymphomas, or Histiocytic Disorders (The Pediatric MATCH Screening Trial) (NCT03155620)	Procedure: Biopsy Procedure: Biospecimen collection Drug: Ensartinib (and 12 more)	Allocation: Nonrandomized Intervention model: Parallel assignment Masking: None (open label) Primary purpose: Screening	Children’s Hospital of Alabama, Birmingham, AL, USA Cardon Children’s Medical Center, Mesa, AZ, USA Phoenix Children’s Hospital, Phoenix, AZ, USA (and 143 more)
Palbociclib in Treating Patients With Relapsed or Refractory Rb Positive Advanced Solid Tumors, Non-Hodgkin Lymphoma, or Histiocytic Disorders With Activating Alterations in Cell Cycle Genes (A Pediatric MATCH Treatment Trial) (NCT03526250)	Other: Laboratory biomarker analysis Drug: Palbociclib Other: Pharmacological study	Intervention model: Single-group assignment Masking: None (open label) Primary purpose: Treatment	Children’s Hospital of Alabama, Birmingham, AL, USA Cardon Children’s Medical Center, Mesa, AZ, USA Phoenix Children’s Hospital, Phoenix, AZ, USA (and 93 more)
Ulixertinib in Treating Patients With Advanced Solid Tumors, Non-Hodgkin Lymphoma, or Histiocytic Disorders With MAPK Pathway Mutations (A Pediatric MATCH Treatment Trial) (NCT03698994)	Other: Pharmacokinetic study Drug: Ulixertinib	Intervention model: Single-group assignment Masking: None (open label) Primary purpose: Treatment	Children’s Hospital of Alabama, Birmingham, AL, USA Cardon Children’s Medical Center, Mesa, AZ, USA Arkansas Children’s Hospital, Little Rock, AR, USA (and 94 more)
Olaparib in Treating Patients With Relapsed or Refractory Advanced Solid Tumors, Non-Hodgkin Lymphoma, or Histiocytic Disorders With Defects in DNA Damage Repair Genes (A Pediatric MATCH Treatment Trial) (NCT03233204)	Drug: Olaparib	Intervention model: Single-group assignment Masking: None (open label) Primary purpose: Treatment	Children’s Hospital of Alabama, Birmingham, AL, USA Cardon Children’s Medical Center Mesa, AZ, USA Arkansas Children’s Hospital, Little Rock, AR, USA (and 102 more)
Erdafitinib in Treating Patients With Relapsed or Refractory Advanced Solid Tumors, Non-Hodgkin Lymphoma, or Histiocytic Disorders With FGFR Mutations (A Pediatric MATCH Treatment Trial) (NCT03210714)	Drug: Erdafitinib Other: Laboratory biomarker analysis Other: Pharmacological study	Intervention model: Single-group assignment Masking: None (open label) Primary purpose: Treatment	Children’s Hospital of Alabama, Birmingham, AL, USA Cardon Children’s Medical Center, Mesa, AZ, USA Arkansas Children’s Hospital, Little Rock, AR, USA (and 103 more)
PI3K/mTOR Inhibitor LY3023414 in Treating Patients With Relapsed or Refractory Advanced Solid Tumors, Non-Hodgkin Lymphoma, or Histiocytic Disorders With TSC or PI3K/MTOR Mutations (A Pediatric MATCH Treatment Trial) (NCT03213678)	Other: Laboratory biomarker analysis Other: Pharmacological study Drug: Samotolisib	Intervention model: Single-group assignment Masking: None (open label) Primary purpose: Treatment	Children’s Hospital of Alabama, Birmingham, AL, USA Cardon Children’s Medical Center, Mesa, AZ, USA Arkansas Children’s Hospital, Little Rock, AR, USA (and 104 more)
Tazemetostat in Treating Patients With Relapsed or Refractory Advanced Solid Tumors, Non-Hodgkin Lymphoma, or Histiocytic Disorders With EZH2, SMARCB1, or SMARCA4 Gene Mutations (A Pediatric MATCH Treatment Trial) (NCT03213665)	Other: Laboratory biomarker analysis Drug: Tazemetostat	Intervention model: Single-group assignment Masking: None (open label) Primary purpose: Treatment	Children’s Hospital of Alabama, Birmingham, AL, USA Cardon Children’s Medical Center, Mesa, AZ, USA Phoenix Children’s Hospital, Phoenix, AZ, USA (and 106 more)
Larotrectinib in Treating Patients With Relapsed or Refractory Advanced Solid Tumors, Non-Hodgkin Lymphoma, or Histiocytic Disorders With NTRK Fusions (A Pediatric MATCH Treatment Trial) (NCT03213704)	Drug: Larotrectinib Drug: Larotrectinib sulfate	Intervention model: Single-group assignment Masking: None (open label) Primary purpose: Treatment	Children’s Hospital of Alabama, Birmingham, AL, USA Cardon Children’s Medical Center, Mesa, AZ, USA Arkansas Children’s Hospital, Little Rock, AR, USA (and 105 more)
CD34+ (Non-Malignant) Stem Cell Selection for Patients Receiving Allogeneic Stem Cell Transplantation (NCT01966367)	Biological: CD34 stem cell selection therapy	Intervention model: Single-group assignment Masking: None (open label) Primary purpose: Treatment	NewYork-Presbyterian Morgan Stanley Children’s Hospital, Columbia University, New York, NY, USA
Vemurafenib in Treating Patients With Relapsed or Refractory Advanced Solid Tumors, Non-Hodgkin Lymphoma, or Histiocytic Disorders With BRAF V600 Mutations (A Pediatric MATCH Treatment Trial) (NCT03220035)	Other: Laboratory biomarker analysis Drug: Vemurafenib	Intervention model: Single-group assignment Masking: None (open label) Primary purpose: Treatment	Children’s Hospital of Alabama, Birmingham, AL, USA Cardon Children’s Medical Center, Mesa, AZ, USA Arkansas Children’s Hospital, Little Rock, AR, USA (and 103 more)
Allogeneic Hematopoietic Stem Cell Transplant for Patients With Primary Immune Deficiencies (NCT01652092)	Drug: Alemtuzumab 0.3 mg Drug: Cyclophosphamide Drug: Busulfan (and 6 more)	Allocation: Nonrandomized intervention model: Single-group assignment Masking: None (open label) Primary purpose: Treatment	Masonic Cancer Center, University of Minnesota, Minneapolis, MN, USA
Study to Investigate Safety, Pharmacokinetic (PK), Pharmacodynamic (PD) and Clinical Activity of Trametinib in Subjects With Cancer or Plexiform Neurofibromas and Trametinib in Combination With Dabrafenib in Subjects With Cancers Harboring V600 Mutations (NCT02124772)	Drug: Trametinib Drug: Dabrafenib	Allocation: Nonrandomized Masking: None (open label) Primary purpose: Treatment	Novartis Investigative Site, Phoenix, AZ, USA Novartis Investigative Site, San Francisco, CA, USA Novartis Investigative Site, Baltimore, MD, USA (and 13 more)
Pediatric Long-Term Follow-up and Rollover Study (NCT03975829)	Drug: Dabrafenib Drug: Trametinib	Intervention model: Single-group assignment Masking: None (open label) Primary purpose: Treatment	
CD34+ (Malignant) Stem Cell Selection for Patients Receiving Allogenic Stem Cell Transplant (NCT02061800)	Device: CliniMACS CD34+ reagent system (Miltenyi Biotech, Bergisch Gladbach, Germany) Drug: Thiotepa Drug: Cyclophosphamide (and 6 more)	Allocation: Nonrandomized Intervention model: Parallel assignment Masking: None (open label) Primary purpose: Treatment	Columbia University Medical Center, New York, NY, USA

List is current as of October 25, 2019. Adapted from ClinicalTrials.gov

## Data Availability

This report does not contain any data. This section is not applicable.
